# Supplementary material for: Synergism between two BLA-to-BNST pathways for appropriate expression of anxiety-like behaviors in male mice
Source: Nat Commun. 2024 Apr 24;15:3455. doi: 10.1038/s41467-024-47966-2 (PMC11043328; doi:10.1038/s41467-024-47966-2)
Supplement: Supplementary file 1 — Supplementary Information [file 41467_2024_47966_MOESM1_ESM.pdf]

a

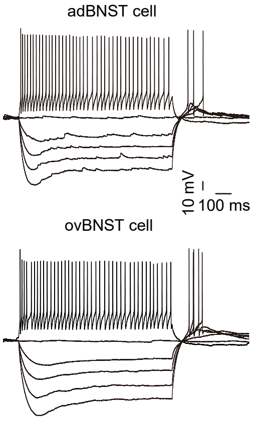

b

	adBNST cells	ovBNST cells	*p*
V_{rest} (mV)	-72.4 ± 0.6	-75.1 ± 1.2	NS
R_{in} (megohms)	561.0 ± 43.9	445 ± 37.6	NS
C_m (pF)	29.4 ± 4.1	53.7 ± 6.1	**
Rheobase (pA)	19.7 ± 2.0	27.0 ± 3.1	NS
AP threshold (mV)	-45.4 ± 0.9	-46.9 ± 0.8	NS
AP amplitude (mV)	77.1 ± 1.8	77.7 ± 0.6	NS
AP half width (ms)	0.81 ± 0.06	0.75 ± 0.04	NS
fAHP (mV)	8.99 ± 0.92	7.18 ± 0.45	NS

Supplementary Fig. 1 The electrophysiological characteristics of the adBNST and ovBNST neurons. a, Representative traces showing electrophysiological response to injection of current with stepwise increase of strength into an adBNST (top) and ovBNST (bottom) neurons. **b**, Quantification of the membrane parameters of adBNST (n = 14 neurons/4 mice) and ovBNST (n = 15 neurons/4 mice) neurons. ***p* < 0.01, multiple t-test. *V*m: resting membrane potential; *R*m: membrane resistance; *C*m: capacitance; AP: action potential; fAHP: fast afterhyperpolarization. All data shown as means ± s.e.m. All data shown as means ± s.e.m. Source data including statistics are provided as a Source Date file.

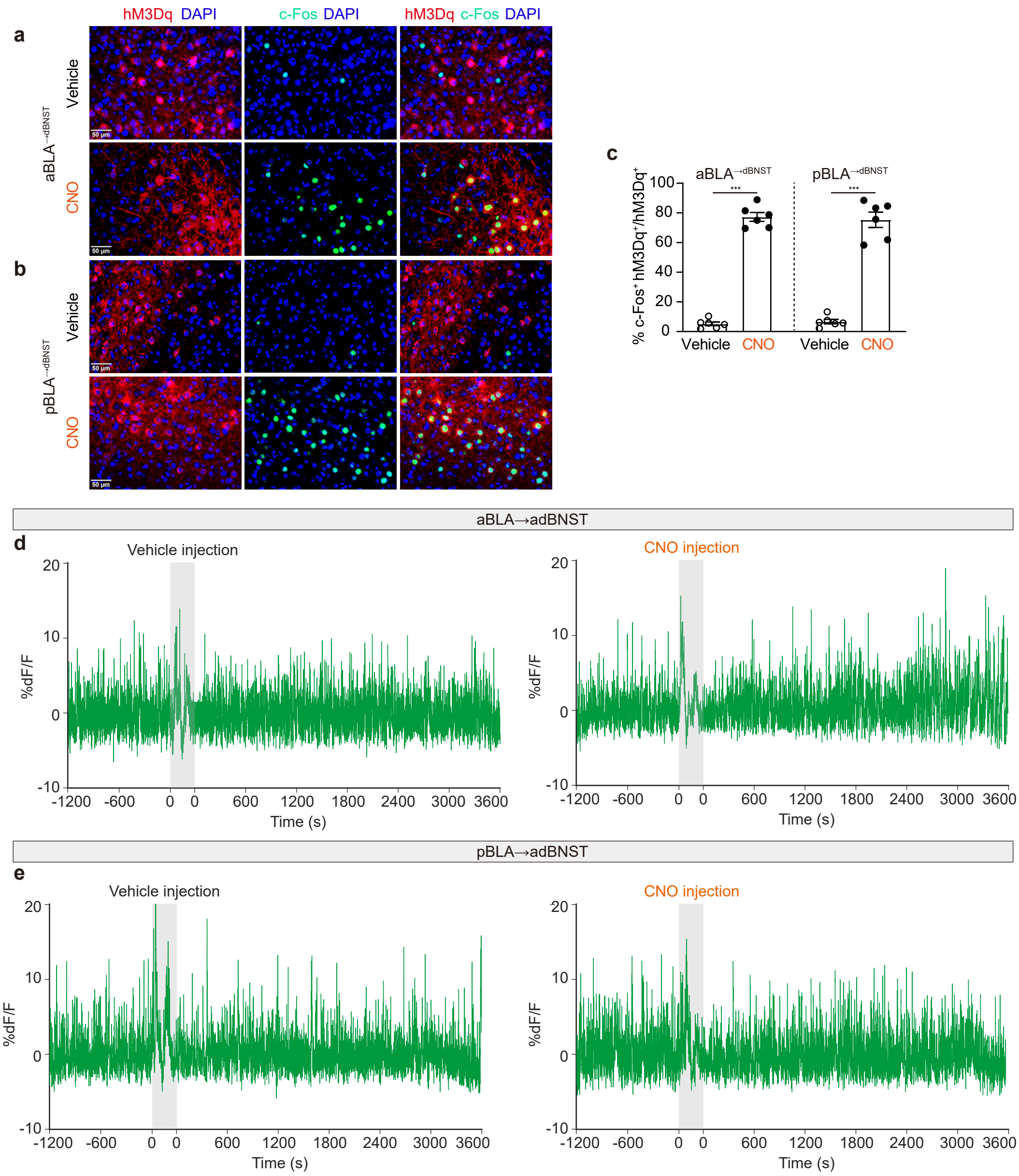

Supplementary Fig. 2 Validation of chemogenetic activation of aBLA$^{\rightarrow dBNST}$ and pBLA$^{\rightarrow dBNST}$ PNs. **a**, Representative images showing c-Fos (green) expression in hM3Dq-positive aBLA$^{\rightarrow dBNST}$ PNs (red) after vehicle (top) or CNO treatment (bottom). Images were merged on the Right. **b**, Same as **a** except that hM3Dq was expressed in pBLA$^{\rightarrow dBNST}$ PNs. **c**, Quantification of the percentage of total hM3Dq PNs expressing c-Fos. n = 6 mice/group, $^{***}p <$ 0.001, multiple t-test. **d**, Representative traces showing the calcium signal in the adBNST neurons before and after vehicle (left) or CNO (right) treatment in mice that expressed hM3Dq in aBLA$^{\rightarrow dBNST}$ PNs **e**, Same as (**d**) except that hM3Dq was expressed in pBLA$^{\rightarrow dBNST}$ PNs. All data shown as means ± s.e.m. Source data including statistics are provided as a Source Date file.

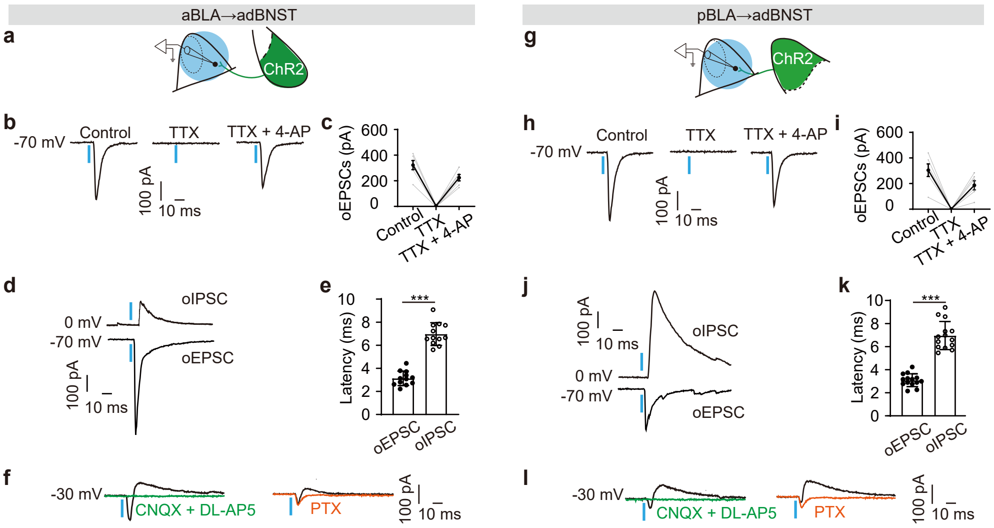

Supplementary Fig. 3 Optogenetic activation of aBLA and pBLA inputs evokes monosynaptic oEPSCs and disynaptic oIPSCs in adBNST neurons. a, Schematic showing light stimulation of aBLA inputs to evoke synaptic response in adBNST neurons. **b**, Representative traces showing oEPSCs when slices were sequentially perfused with ACSF, TTX (1 μM) and TTX (1 μM) + 4-AP (100 μM). Neurons were held at -70 mV. **c**, Summary of the oEPSCs amplitudes as shown in **b**. Data from individual neurons were shown in gray. n = 6 neurons/3 mice; **d**, Representative traces of oEPSCs and oIPSCs in adBNST neurons at -70 and 0 mV, respectively. **e**, Summary of the synaptic latency of oEPSCs and oIPSCs as shown in **d**. n = 12 neurons/4 mice, ***$p < 0.001$, two-tailed paired t-test. **f**, Representative traces showing effects of CNQX (20 μM) and AP5 (50 μM) or PTX (100 μM) on oEPSCs/oIPSCs in adBNST neurons at -30 mV. **g**-**l**, Same as in **a**-**f**, except that pBLA inputs were stimulated. **i**: n = 6 neurons/3 mice; **k**: n = 14 neurons/4 mice, ***$p < 0.001$, two-tailed paired t-test. All data shown as means ± s.e.m. Source data including statistics are provided as a Source Date file.

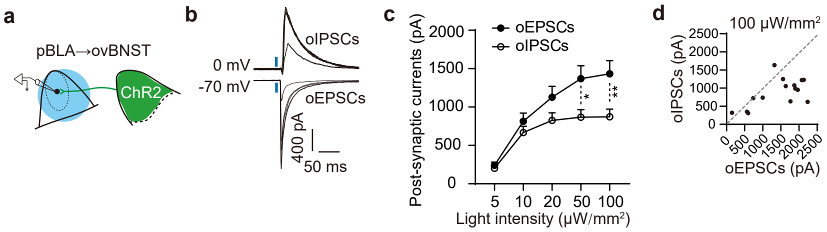

Supplementary Fig. 4 pBLA inputs activation evoked robust oEPSCs and oIPSCs in ovBNST cells. a, Schematic showing light stimulation of pBLA inputs to evoke synaptic response in ovBNST neurons. **b**, Representative traces of oEPSCs and oIPSCs in ovBNST neuron at -70 and 0 mV, respectively. **c**, Summary of oEPSCs and oIPSCs amplitudes against the increasing power of light stimuli as shown in **b**. n=15 neurons/5 mice. *$p < 0.05$, **$p < 0.01$, two-way ANOVA with Bonferroni's multiple comparisons test. **d**, Distribution of the oEPSCs and oIPSCs amplitudes in each recorded ovBNST cells when the light power was set at 100 μW/mm^2. All data shown as means ± s.e.m. Source data including statistics are provided as a Source Date file.

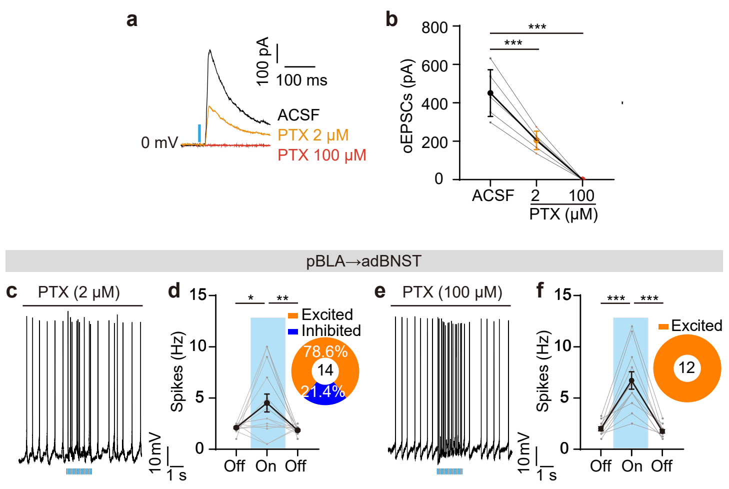

Supplementary Fig. 5 Blocking GABAergic transmission reverses pBLA-evoked suppression of adBNST neuronal activity. a, Representative traces showing oIPSCs when slice was sequentially perfused with ACSF, PTX (2 μM) and PTX (100 μM). Neurons were held at -0 mV. **b**, Summary of the oIPSCs amplitudes as shown in **a**. Data from individual neurons were shown in gray. n = 6 neurons/3 mice. $^{**}p < 0.01$, $^{***}p < 0.001$, one-way ANOVA with Bonferroni's multiple comparisons test. **c**, Representative trace showing adBNST neuronal firing before, during and after pBLA inputs activation in the presence of PTX (2 μM). **d**, Summary of the spike frequency as shown in **c**. Data from individual neurons were shown in gray. n = 14 neurons/4 mice; $^{*}p < 0.05$, $^{**}p < 0.01$, one-way ANOVA with Bonferroni's multiple comparisons test. **e-f**, Same as in **c-d**, except that the concentration of PTX was 100 μM. n = 12 neurons/4 mice; $^{***}p < 0.001$, one-way ANOVA with Bonferroni's multiple comparisons test. All data shown as means ± s.e.m. Source data including statistics are provided as a Source Date file.

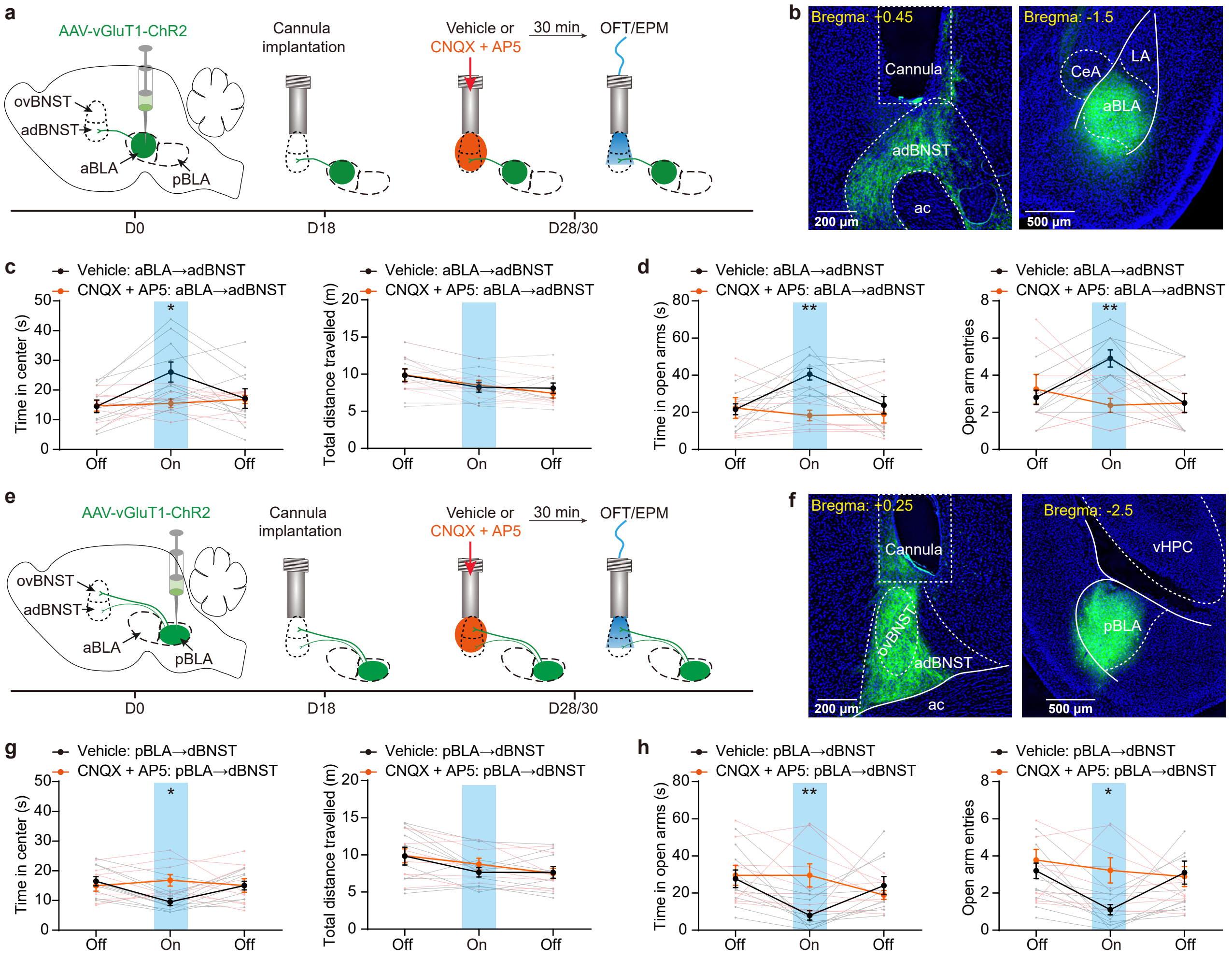
a
AAV-vGluT1-ChR2
ovBNST
adBNST
aBLA
pBLA
Cannula implantation
Vehicle or
CNQX + AP5
30 min
OFT/EPM
D0
D18
D28/30
b
Bregma: +0.45
Cannula
adBNST
ac
200 μm
Bregma: -1.5
CeA
LA
aBLA
500 μm
c
Vehicle: aBLA→adBNST
CNQX + AP5: aBLA→adBNST
Time in center (s)
Total distance travelled (m)
Off
On
Off
d
Time in open arms (s)
Open arm entries
e
f
Bregma: +0.25
ovBNST
Bregma: -2.5
vHPC
pBLA
g
Vehicle: pBLA→dBNST
CNQX + AP5: pBLA→dBNST
h

Supplementary Fig. 6 The direct aBLA and pBLA inputs onto dBNST are required for anxiety regulation of BLA→dBNST pathways. **a**, Experimental procedures for investigating the necessity of aBLA inputs onto dBNST in regulating anxiety-like behaviors in mice. OFT, open field test, EPM, elevated plus maze. **b**, Left: Representative image showing the axonal terminals in dBNST from aBLA PNs. The position of cannula was marked with the dashed lines. Right: ChR2 expression in aBLA PNs. This experiment was repeated 18 times with similar results. **c**, Summary of the time in center and total distance travelled in OFT before, during and after light stimulation of ChR2-expressing aBLA projections in mice pretreated with vehicle or CNQX+AP5. Data from individual mice were shown in gray. n = 10 (vehicle), 8 (CNQX+AP5) mice. $^{*}p < 0.05$, two-way ANOVA with Bonferroni's multiple comparisons test. **d**, Summary of the time in open arms and open-arm entries in EPM before, during and after light stimulation of ChR2-expressing aBLA projections in mice pretreated with vehicle or CNQX+AP5. Data from individual mice were shown in gray. $^{**}p < 0.01$, two-way ANOVA with Bonferroni's multiple comparisons test. **e**-**h**, same as in **a**-**d**, except that ChR2 was expressed in pBLA PNs. n = 10 (vehicle), 9 (CNQX+AP5) mice. $^{*}p < 0.05$ and $^{**}p < 0.01$, two-way ANOVA with Bonferroni's multiple comparisons test. All data shown as means ± s.e.m. Source data including statistics are provided as a Source Date file.

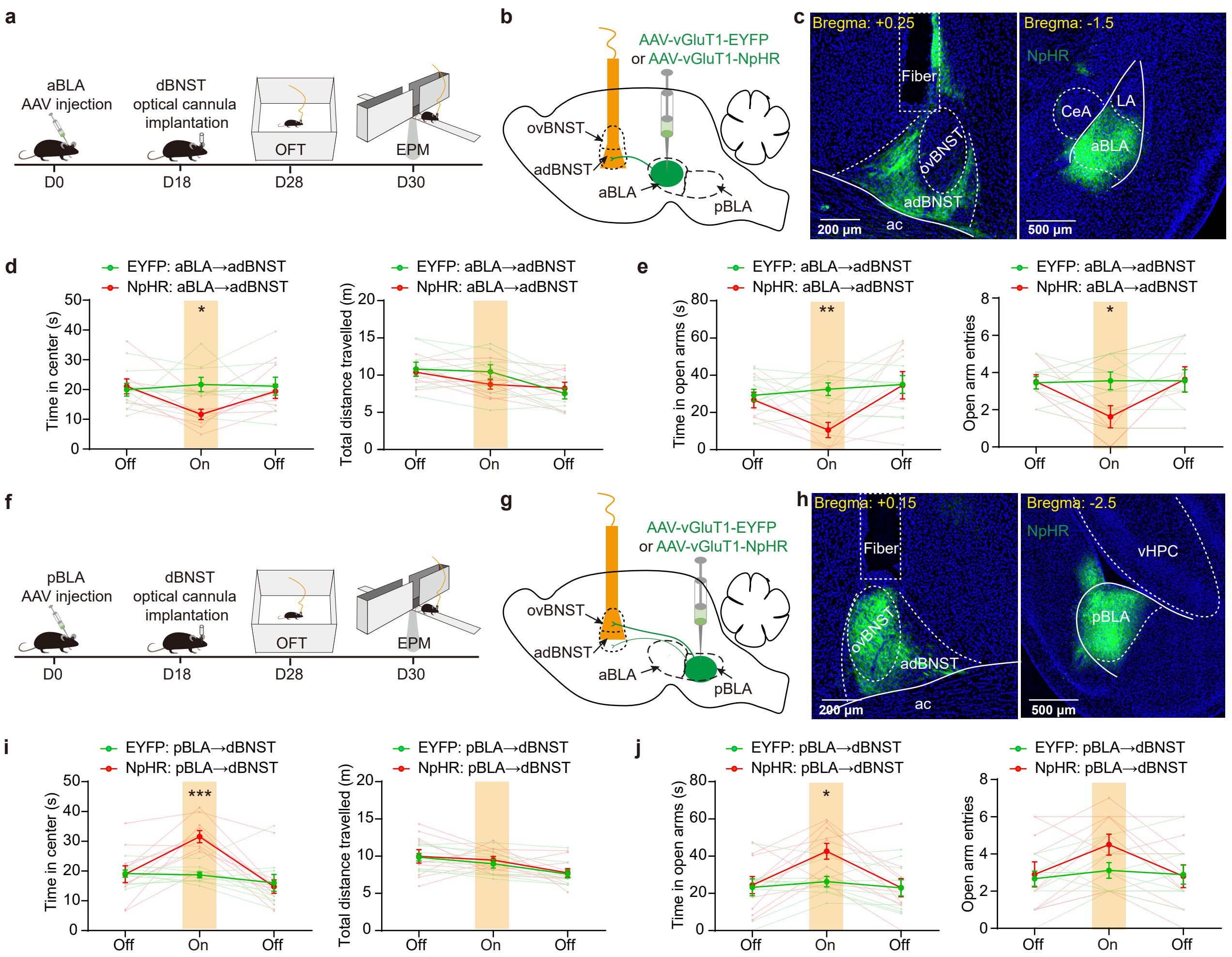
a
aBLA
AAV injection
dBNST
optical cannula
implantation
OFT
EPM
D0
D18
D28
D30
b
AAV-vGluT1-EYFP
or AAV-vGluT1-NpHR
ovBNST
adBNST
aBLA
pBLA
c
Bregma: +0.25
Fiber
ovBNST
adBNST
ac
200 µm
Bregma: -1.5
NpHR
CeA
LA
aBLA
500 µm
d
EYFP: aBLA→adBNST
NpHR: aBLA→adBNST
Time in center (s)
Total distance travelled (m)
Off
On
Off
e
Time in open arms (s)
Open arm entries
f
pBLA
AAV injection
dBNST
optical cannula
implantation
g
h
Bregma: +0.15
Bregma: -2.5
vHPC
pBLA
i
EYFP: pBLA→dBNST
NpHR: pBLA→dBNST
j

Supplementary Fig. 7 Optogenetic inhibition of aBLA→adBNST and pBLA→dBNST pathways oppositely regulates the anxiety-like behaviors in mice. a, Experimental procedures for investigating the effect of optogenetic inhibition of aBLA→adBNST pathway in regulating anxiety-like behaviors. OFT, open field test, EPM, elevated plus maze. **b**, Schematic showing injection of AAV vectors encoding EYFP or NpHR into aBLA and implantation of optical cannula onto adBNST for optogenetic manipulations. **c**, Left: Representative image showing the axonal terminals in dBNST from aBLA PNs. The position of optical cannula was marked with the dashed lines. Right: NpHR expression in aBLA PNs. This experiment was repeated 8 times with similar results. **d**, Summary of the time in center and total distance travelled in OFT before, during and after light stimulation of the EYFP- or NpHR-expressing aBLA projections. Data from individual mice were shown in gray. n = 9 (EYFP), 8 (NpHR) mice. $^{*}p < 0.05$, two-way ANOVA with Bonferroni's multiple comparisons test. **e**, Summary of the time in open arms and open-arm entries in EPM before, during and after light stimulation of EYFP- or NpHR-expressing aBLA projections. Data from individual mice were shown in gray. $^{*}p < 0.05$ and $^{**}p < 0.01$, two-way ANOVA with Bonferroni's multiple comparisons test. **f**-**j**, same as in **a**-**e**, except that NpHR was expressed in pBLA PNs and the optical cannula was implanted onto dBNST. n = 9 (EYFP), 10 (NpHR) mice. $^{*}p < 0.05$ and $^{***}p < 0.001$, two-way ANOVA with Bonferroni's multiple comparisons test. All data shown as means ± s.e.m. Source data including statistics are provided as a Source Date file.

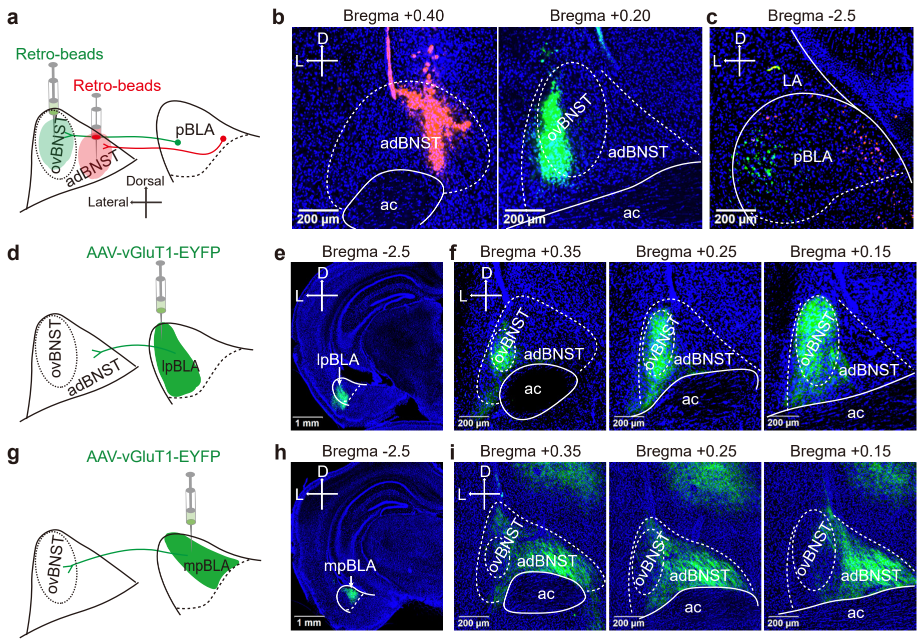

Supplementary Fig. 8 The lpBLA and mpBLA PNs have distinct projection patterns in dBNST. a, Schematic showing injection of red and green retrobeads into adBNST and ovBNST respectively, to retrogradely label the input neurons in the pBLA. **b**, Representative images showing the injection sites in adBNST (left) and ovBNST (right). **c**, Representative image showing the distribution of retrogradely labeled neurons in pBLA. **d**, Schematic showing injection of AAV vectors encoding EYFP into lpBLA to anterogradely labeling their axonal terminals in dBNST. **e**, Representative image showing expression EYFP in lpBLA. **f**, Representative images showing the axonal terminals of lpBLA PNs in dBNST along the anetro-posterior axis. **g-i**, same as in **d-f**, except that the injection site was mpBLA.

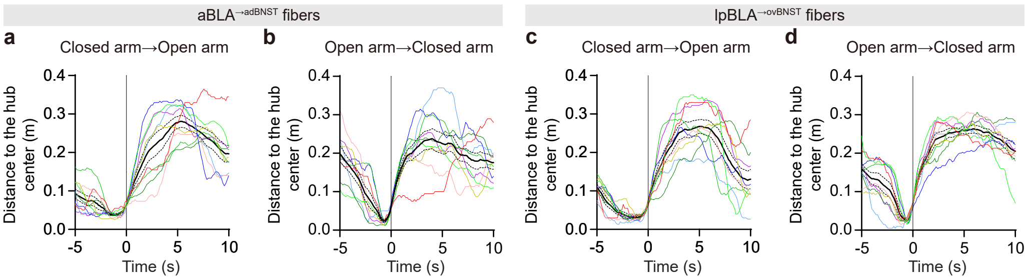

Supplementary Fig. 9 Location of mice during exploring EPM (related to Fig. 7). a-b, The distance between the center of mice to the hub center during recording the calcium signals in the aBLA$^{\rightarrow adBNST}$ fibers in EPM. Data from individual mice were shown in light colors. **c-d,** Same as a-b except that the recordings were made on lpBLA$^{\rightarrow ovBNST}$ fibers. All data shown as means ± s.e.m (s.e.m shown as dashed lines). Source data are provided as a Source Date file.

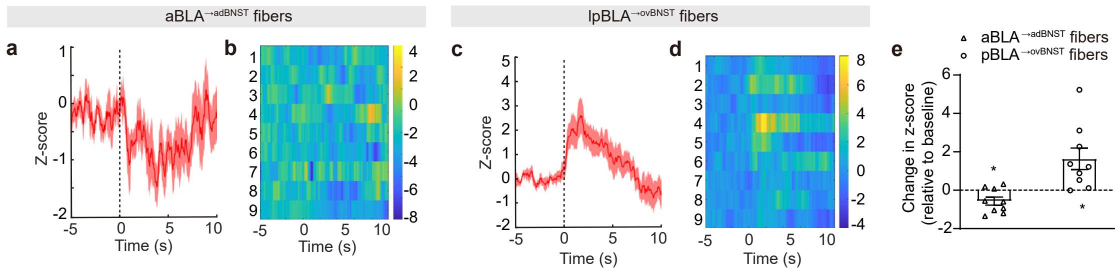

Supplementary Fig. 10 The calcium activity of BLA PN axonal terminals in dBNST (related to Fig. 7). a, Changes of average calcium signals in aBLA$^{\rightarrow adBNST}$ fibers in the mice that partially entered the open arm and returned to the closed arm. n = 9 mice. **b,** Heat maps showing the calcium fluorescence in the aBLA$^{\rightarrow adBNST}$ terminals of individual mice (2-3 trails for each) in **a**. **c-d**. Same as in **a-b** except that the calcium signals were recorded in lpBLA$^{\rightarrow ovBNST}$ fibers. n = 9 mice. 2-3 trails for each. **e**, Summary of the changes of the average calcium signals in BLA$^{\rightarrow dBNST}$ fibers during 0 to 10 s relative to the baseline (-5 to 0 s) as shown in a-d. Please not that time 0 s was set as when the head of mice entering the open arm. *$p < 0.05$, two tailed one sample t test. All data shown as means ± s.e.m. Source data including statistics are provided as a Source Date file.

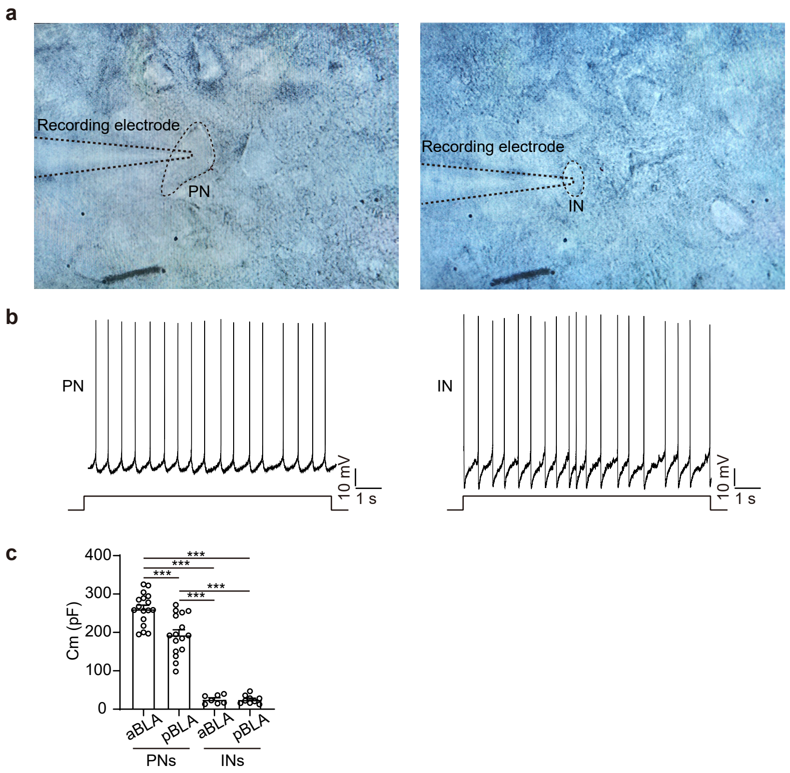

Supplementary Fig. 11 Morphological and electrophysiological characterization of BLA PNs and interneurons (INs). a, Representative images showing the recording of PN and IN in BLA. The dashed lines show the recording pipette and the morphology of the recorded PN (left) and IN (right) **b**, Representative traces showing the firing of PN and IN in response to the injection of a depolarizing current of approximately 100 pA for PN and 15 pA for IN. **c**, Quantification of the membrane capacitance (*C*m) of aBLA and pBLA PNs and INs. PNs: n = 17 (aBLA), 16 (pBLA) neurons; INs: n = 7 (aBLA), 9 (pBLA) neurons. ***$p < 0.001$, one-way ANOVA with Bonferroni's multiple comparisons test. All data shown as means ± s.e.m. Source data including statistics are provided as a Source Date file.
