# Supplementary material for: Synergism between two BLA-to-BNST pathways for appropriate expression of anxiety-like behaviors in male mice
Source: Nat Commun. 2024 Apr 24;15:3455. doi: 10.1038/s41467-024-47966-2 (PMC11043328; doi:10.1038/s41467-024-47966-2)
Supplement: Supplementary file 3 — Reporting Summary [file 41467_2024_47966_MOESM3_ESM.pdf]

nature portfolio

Corresponding author(s): Ren-Wen Han; Bing-Xing Pan

Last updated by author(s): Mar 26, 2024

Reporting Summary

Nature Portfolio wishes to improve the reproducibility of the work that we publish. This form provides structure for consistency and transparency in reporting. For further information on Nature Portfolio policies, see our Editorial Policies and the Editorial Policy Checklist.

Statistics

For all statistical analyses, confirm that the following items are present in the figure legend, table legend, main text, or Methods section.

n/a	Confirmed	
☐	☒	The exact sample size (*n*) for each experimental group/condition, given as a discrete number and unit of measurement
☐	☒	A statement on whether measurements were taken from distinct samples or whether the same sample was measured repeatedly
☐	☒	The statistical test(s) used AND whether they are one- or two-sided<br>*Only common tests should be described solely by name; describe more complex techniques in the Methods section.*
☐	☒	A description of all covariates tested
☐	☒	A description of any assumptions or corrections, such as tests of normality and adjustment for multiple comparisons
☐	☒	A full description of the statistical parameters including central tendency (e.g. means) or other basic estimates (e.g. regression coefficient) AND variation (e.g. standard deviation) or associated estimates of uncertainty (e.g. confidence intervals)
☐	☒	For null hypothesis testing, the test statistic (e.g. F, t, r) with confidence intervals, effect sizes, degrees of freedom and P value noted<br>*Give P values as exact values whenever suitable.*
☒	☐	For Bayesian analysis, information on the choice of priors and Markov chain Monte Carlo settings
☒	☐	For hierarchical and complex designs, identification of the appropriate level for tests and full reporting of outcomes
☒	☐	Estimates of effect sizes (e.g. Cohen's *d*, Pearson's *r*), indicating how they were calculated

Our web collection on statistics for biologists contains articles on many of the points above.

Software and code

Policy information about availability of computer code

Data collection	Electrophysiologica data were collected using HEKA's PatchMaster software (version 2.53). Behavioral data were collected using video tracking system (Med Associates Inc., Farifax, VT). Calcium signals were collected by the fiber photometry system (Thinker Tech Nanjing Biotech Limited Co., Ltd). Immunofluorescence images were taken by using high-throughput microscope (Olympus VS120, Tokyo, Japan) and scanning laser microscope (Olympus FV1000, Tokyo, Japan).
Data analysis	Electrophysiological data were analyzed by Origin 8.5. Behavioral data were analyzed by video-tracking system (Med Associates Inc., Farifax, VT). Calcium signals were analyzed using MATLAB-based software affiliated with the fiber photometry system (Thinker Tech Nanjing Biotech Limited Co., Ltd). All statistical analysis were performed by GraphPad Prism 7 (Graphpad Software).

For manuscripts utilizing custom algorithms or software that are central to the research but not yet described in published literature, software must be made available to editors and reviewers. We strongly encourage code deposition in a community repository (e.g. GitHub). See the Nature Portfolio guidelines for submitting code & software for further information.

Data

Policy information about availability of data

All manuscripts must include a data availability statement. This statement should provide the following information, where applicable:

- Accession codes, unique identifiers, or web links for publicly available datasets
- A description of any restrictions on data availability
- For clinical datasets or third party data, please ensure that the statement adheres to our policy

Source data are provided with the paper.

Research involving human participants, their data, or biological material

Policy information about studies with human participants or human data. See also policy information about sex, gender (identity/presentation), and sexual orientation and race, ethnicity and racism.

Reporting on sex and gender	N/A
Reporting on race, ethnicity, or other socially relevant groupings	N/A
Population characteristics	N/A
Recruitment	N/A
Ethics oversight	N/A

Note that full information on the approval of the study protocol must also be provided in the manuscript.

Field-specific reporting

Please select the one below that is the best fit for your research. If you are not sure, read the appropriate sections before making your selection.

☒ Life sciences ☐ Behavioural & social sciences ☐ Ecological, evolutionary & environmental sciences

For a reference copy of the document with all sections, see nature.com/documents/nr-reporting-summary-flat.pdf

Life sciences study design

All studies must disclose on these points even when the disclosure is negative.

Sample size	Sample sizes were determined based on previous experience and related literature.
Data exclusions	Pre-established exclusion criteria for exprimenta data points included lack of accurate stereotaxic targeting and/or viral expression based on post-mortem brain tissue analyses. No outliers were excluded in this manuscript.
Replication	All measurements were taken from distinct samples.
Randomization	For behavioral experiments, mice were randomly selected to initially receive either the vehicle or drugs.
Blinding	For the physiological experiments, investigators were blinded to group allocation during data collection and analysis. The behavioral data were collected and analyzed using computer software in an unbiased manner, rendering blinding unnecessary.

Reporting for specific materials, systems and methods

We require information from authors about some types of materials, experimental systems and methods used in many studies. Here, indicate whether each material, system or method listed is relevant to your study. If you are not sure if a list item applies to your research, read the appropriate section before selecting a response.

Materials & experimental systems

n/a	Involved in the study
☐	☒ Antibodies
☒	☐ Eukaryotic cell lines
☒	☐ Palaeontology and archaeology
☐	☒ Animals and other organisms
☒	☐ Clinical data
☒	☐ Dual use research of concern
☒	☐ Plants

Methods

n/a	Involved in the study
☒	☐ ChIP-seq
☒	☐ Flow cytometry
☒	☐ MRI-based neuroimaging

Antibodies

Antibodies used	Primary antibody: c-Fos (9F6) Rabbit monoclonal antibody (1:500; Cell signaling, 2250s); Fuorescent secondary antibody: donkey anti-rabbit Alexa Fluor™ 488 (1:1000; Invitrogen, A21206).
Validation	https://www.cellsignal.cn/products/primary-antibodies/c-fos-9f6-rabbit-mab/2250?site-search-type=Products&N=4294956287&Ntt=c-fos&fromPage=plp https://www.thermofisher.cn/cn/zh/antibody/product/Donkey-anti-Rabbit-IgG-H-L-Highly-Cross-Adsorbed-Secondary-Antibody-Polyclonal/A-21206

Animals and other research organisms

Policy information about studies involving animals; ARRIVE guidelines recommended for reporting animal research, and Sex and Gender in Research

Laboratory animals	Male C57BL/6J mice (5–10 weeks) were used for all experiments.
Wild animals	The study did not involve wild animals.
Reporting on sex	All data were collected from male animals.
Field-collected samples	The study did not involve data collected from the field.
Ethics oversight	All experimental procedures were conducted in accordance with the guidelines of the National Institutes of Health and approved by the Institutional Animal Care and Use Committee of Nanchang University.

Note that full information on the approval of the study protocol must also be provided in the manuscript.
